# Prophylactic intestinal resection following carbon-ion radiotherapy for locally recurrent rectal cancer: a case report

**DOI:** 10.1093/jscr/rjaf788

**Published:** 2025-10-02

**Authors:** Takaki Furuyama, Hirotoshi Takiyama, Ito Kondo, Megumu Enjyoji, Makoto Hinokida, Yusuke Tatsutomi, Kunihiko Nakazawa, Shigeru Yamada, Yoshio Ushirokoji

**Affiliations:** Department of Surgery, Tokyo Kyosai Hospital, 2-3-8 Nakameguro, Meguro-ku, Tokyo 153-8934, Japan; Department of Radiation Oncology, QST Hospital, National Institute for Quantum Science and Technology, 4-9-1 Anagawa, Inage-ku, Chiba 263-8555, Japan; Department of Surgery, Tokyo Kyosai Hospital, 2-3-8 Nakameguro, Meguro-ku, Tokyo 153-8934, Japan; Department of Surgery, JA Toride Medical Center, 2-1-1 Hongo, Toride-city, Ibaraki 302-0022, Japan; Department of Surgery, Tokyo Kyosai Hospital, 2-3-8 Nakameguro, Meguro-ku, Tokyo 153-8934, Japan; Department of Surgery, Tokyo Kyosai Hospital, 2-3-8 Nakameguro, Meguro-ku, Tokyo 153-8934, Japan; Department of Surgery, Tokyo Kyosai Hospital, 2-3-8 Nakameguro, Meguro-ku, Tokyo 153-8934, Japan; Department of Radiation Oncology, QST Hospital, National Institute for Quantum Science and Technology, 4-9-1 Anagawa, Inage-ku, Chiba 263-8555, Japan; Department of Surgery, Tokyo Kyosai Hospital, 2-3-8 Nakameguro, Meguro-ku, Tokyo 153-8934, Japan

**Keywords:** locally recurrent rectal cancer, carbon-ion radiotherapy, prophylactic resection

## Abstract

Treatment of locally recurrent rectal cancer (LRRC) after surgery is often complex and challenging. A 52-year-old man received emergency surgery (Hartmann’s procedure) for bowel perforation caused by a huge sigmoid colon cancer, followed by treatment for concurrent advanced lower rectal cancer with neoadjuvant chemoradiotherapy and abdominoperineal resection. A solitary lung metastasis emerged afterwards, and was surgically removed. However, the patient developed LRRC in front of the sacrum. As surgical resection for the local recurrence was considered too invasive, carbon-ion radiotherapy (CIRT) was performed as radical local therapy. Because the surrounding intestine was highly adherent to the tumor and there was a high risk of developing an ulcer, the intestine was prophylactically resected. The patient has remained relapse-free for 2 years and 6 months since the most recent surgery. CIRT for LRRC appears to represent a useful therapeutic option in combination with prophylactic intestinal resection.

## Introduction

Locally recurrent rectal cancer (LRRC) severely affects both prognosis and quality of life. Although surgical resection is the only curative treatment for LRRC, curative surgery for LRRC is highly invasive. Favorable clinical outcomes for LRRC have recently been reported with carbon-ion radiotherapy (CIRT), even in previously irradiated patients. However, CIRT for LRRC is inapplicable when the recurrent tumor is attached to radiosensitive organs such as the bowel and bladder. We report a case of LRRC successfully treated with CIRT followed by prophylactic resection of the irradiated small intestine.

## Case report

**Figure 1:**
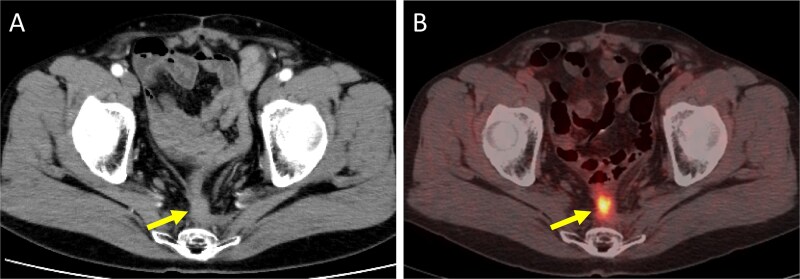
Imaging findings at the time of local recurrence diagnosis. (A) Contrast-enhanced CT. (B) Positron emission tomography-CT. Arrows indicate the recurrent lesion.

**Figure 2:**
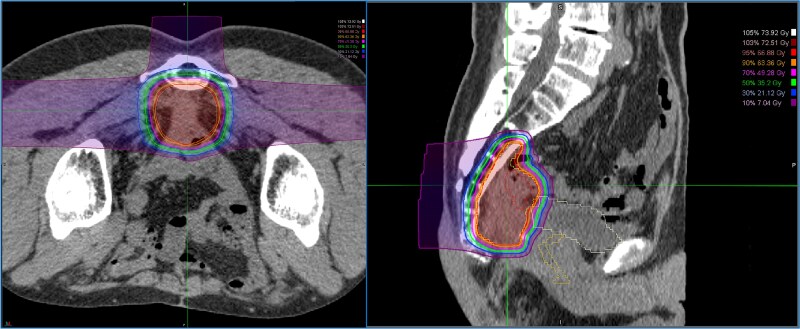
Dose distribution for carbon ion radiotherapy. The irradiation field includes the recurrent lesion, the anterior surface of the sacrum, and the adjacent small intestine.

**Figure 3:**
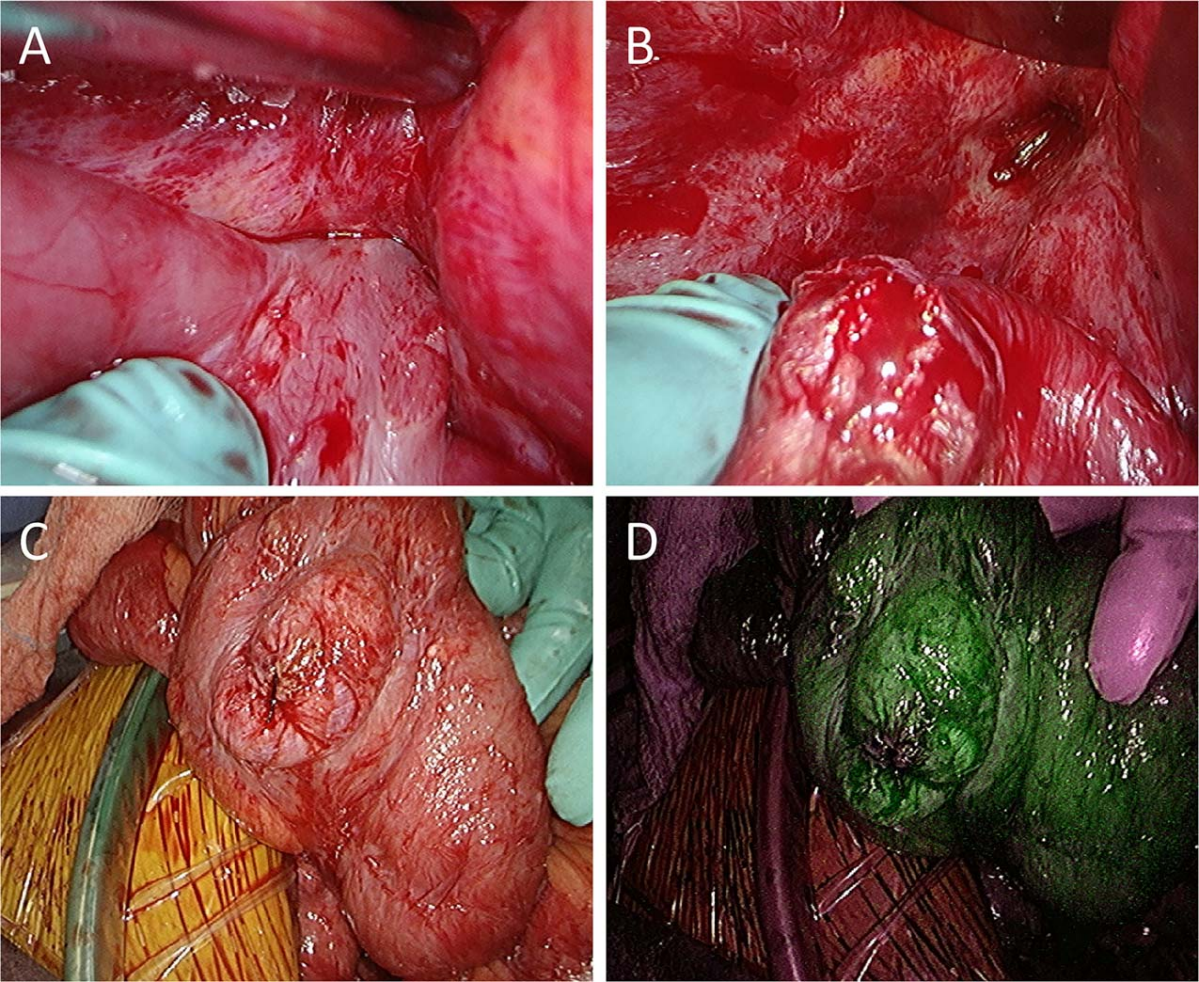
Intraoperative findings. (A) The small intestine is adherent to the locally recurrent lesion at the pelvic floor. (B) The adhesion is successfully separated without injury of the small intestine. (C) The serosal surface of the dissected small intestine shows no gross abnormalities. (D) ICG fluorescence imaging also reveals no evidence of decreased blood flow in the small intestine.

**Figure 4:**
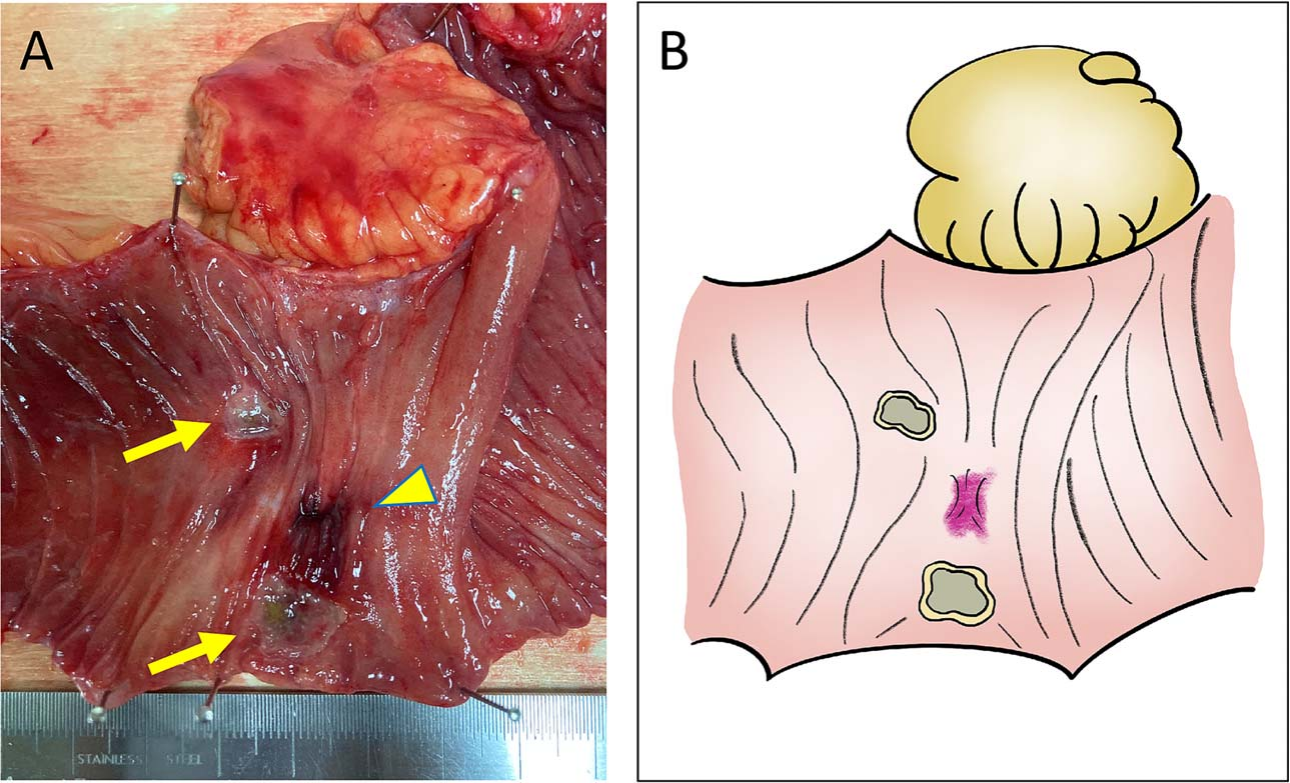
Macroscopic findings. (A) Ulcer formation on the mucosal surface caused by CIRT (arrows, ulcers; arrow head, adhesion site with the recurrent lesion). (B) Illustration of the macroscopic image.

**Figure 5:**
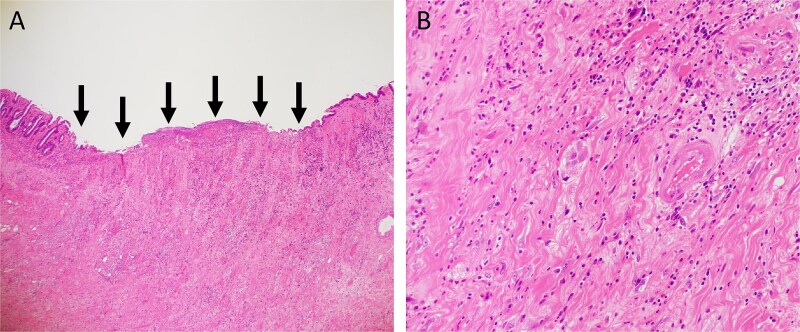
Hematoxylin and eosin staining. (A) Low-power view (magnification, ×40). Arrows indicate the ulcer base. (B) High-power view (magnification, ×200).

A 52-year-old man underwent emergency Hartmann’s procedure for peritonitis caused by perforated sigmoid colon cancer. The tumor was staged as pT4b N0 M0 with no lymphovascular invasion. Postoperative examination revealed synchronous advanced lower rectal cancer. Systemic chemotherapy with mFOLFOX6 plus bevacizumab was administered, followed by neoadjuvant chemoradiotherapy and abdominoperineal resection. Pathological examination confirmed pT3 N0 M0 disease, with no lymphovascular invasion. A total of 2 years and 4 months after the initial surgery, a solitary lung metastasis appeared and was surgically resected. Three years after the initial surgery, imaging revealed LRRC on the anterior surface of the sacrum (Fig. 1). Given the history of multiple laparotomies and treatment for distant metastases, surgical resection was considered inappropriate. After thorough consultation with a CIRT facility and the patient, a plan was made to perform CIRT followed by prophylactic resection of the adjacent irradiated small intestine that was thought to be at a high risk of causing ulcers and perforation. CIRT was administered using carbon ion beams at a dose of 70.4 Gy (relative biological effectiveness weighted dose) in 16 fractions over 4 weeks (Fig. 2). Three months after completion of CIRT, the irradiated small intestine, including a 30-cm segment adherent to the recurrent site, was prophylactically resected (Fig. 3A and B). The serosal surface appeared intact (Fig. 3C), and observation using indocyanine green (ICG) fluorescence imaging indicated good blood flow (Fig. 3D). However, the mucosal surface showed ulcer formation near the adhesion site (Fig. 4). Histopathologically, the ulcerated areas showed necrosis at the ulcer base surface and transmural infiltration of inflammatory cells (Fig. 5). The postoperative course was uneventful, and the patient has remained recurrence-free for 2.5 years.

## Discussion

Despite curative-intent surgery, 5%–10% of rectal cancer patients develop LRRC [1, 2]. Surgical resection offers the best chance of disease control and cure [3], and the 5-year overall survival (OS) rate has been reported as 37%–43% [4–6]. Although achieving R0 resection is associated with improved outcomes in LRRC [4, 7], the risk of incomplete resection remains considerable, and rates of R0 resection have been reported as 50%–60% [4, 5, 7–9]. In addition, extended radical multi-visceral resections such as sacrectomy, total cystectomy, and total abdominal hysterectomy are often required to achieve R0 resection, which are associated with a high morbidity rate (42%–66%) [5, 6, 8].

In recent years, CIRT has emerged as a treatment option for LRRC. CIRT shows two unique characteristics. First, a high dose of energy is delivered with precise localization to the targeted area. Carbon-ion beams provide a unique depth dose distribution profile characterized by the Bragg peak. Most of the energy is deposited at the specific planned depth (called the spread-out Bragg peak; SOBP) in a body so that the dose delivered before and further than the depth of the target is relatively low. The second characteristic is the strong cell-killing activity against cancers. Conventional X-ray therapy primarily causes indirect DNA damage, typically resulting in single-strand breaks. In contrast, CIRT acts directly on DNA, causing double-strand breaks more frequently, that are significantly more difficult to repair than single-strand breaks, resulting in high biological effect. In a phase II trial of 156 LRRC patients, CIRT achieved 5-year local control and OS rates of 88% and 59%, respectively [10]. In 2019, the Japan Carbon-ion Radiation Oncology Study Group (J-CROS) reported similar outcomes in 224 patients, with acceptable toxicity profiles [11]. In addition, CIRT can be administered to patients who have previously received X-ray therapy in the pelvic region [12].

Gastrointestinal ulceration and perforation are among the most serious late toxicities of CIRT. When the radiation dose to the adjacent intestinal tract exceeds 60 Gy, perforation has been reported to occur with a frequency of 19% after 9 months or more post-irradiation [13, 14]. Pre-CIRT spacer placement surgery to physically separate the tumor and bowel is one of the useful methods [15]. However, spacer placement is not recommended when the tumor is in close proximity to or directly invades the gastrointestinal tract, as the risk of cancer seeding and infection due to gastrointestinal injury is high.

In this case, toxicity to the small intestine adjacent to the local recurrence would be inevitable with CIRT. Although placement of a spacer was also considered, concerns regarding the dissemination of cancer cells, intra-abdominal adhesions from multiple previous surgeries, the risk of spacer infection due to bowel perforation, and the lack of tissues such as peritoneum or omentum to cover the placed spacer made this option unfavorable. Consequently, the strategy was chosen to perform CIRT followed by prophylactic resection of the adjacent small intestine. Considering that problematic ulcers and perforations often occur 9–12 months after CIRT, the prophylactic resection surgery was carried out 3 months after CIRT. During surgery the serosal surface of the irradiated small intestine appeared normal, and blood flow was confirmed to be good using ICG fluorescence imaging. However, examination of the resected specimen revealed ulceration already present on the mucosal surface, suggesting that delayed perforation would have eventually occurred. The prophylactic resection in this case enabled timely and definitive administration of CIRT and may serve as a potential treatment option, particularly for cases of LRRC that are considered unsuitable for CIRT due to difficulties in spacer placement.

## Conclusions

CIRT combined with prophylactic intestinal resection can represent a viable therapeutic option for LRRC. Prophylactic resection of the irradiated bowel may expand the indications for CIRT in the treatment of LRRC.
